# Toxicological Characterization and Phospholipase D Activity of the Venom of the Spider *Sicarius thomisoides*

**DOI:** 10.3390/toxins12110702

**Published:** 2020-11-06

**Authors:** Tomás Arán-Sekul, Ivanka Perčić-Sarmiento, Verónica Valencia, Nelly Olivero, José M. Rojas, Jorge E. Araya, Andrés Taucare-Ríos, Alejandro Catalán

**Affiliations:** 1 Laboratorio de Parasitología Molecular, Departamento de Tecnología Médica, Facultad de Ciencias de la Salud, Universidad de Antofagasta, Antofagasta 1270300, Chile; tomas.aran@uantof.cl (T.A.-S.); ivanka.percic.sarmiento@ua.cl (I.P.-S.); veronica.valencia.garcia@ua.cl (V.V.); n.olivero.morgado@gmail.com (N.O.); jose.rojas@uantof.cl (J.M.R.); jorge.araya@uantof.cl (J.E.A.); 2 Facultad de Ciencias, Universidad Arturo Prat, Iquique 1110939, Chile; antaucar@unap.cl; 3 Centro de Investigación en Medio Ambiente (CENIMA), Universidad Arturo Prat, Iquique 1110939, Chile

**Keywords:** *Sicarius thomisoides*, phospholipase D, venom toxicity

## Abstract

Envenomation by *Loxosceles* spiders (*Sicariidae* family) has been thoroughly documented. However, little is known about the potential toxicity of members from the *Sicarius* genus. Only the venom of the Brazilian *Sicarius ornatus* spider has been toxicologically characterized. In Chile, the *Sicarius thomisoides* species is widely distributed in desert and semidesert environments, and it is not considered a dangerous spider for humans. This study aimed to characterize the potential toxicity of the Chilean *S. thomisoides* spider. To do so, specimens of *S. thomisoides* were captured in the Atacama Desert, the venom was extracted, and the protein concentration was determined. Additionally, the venoms were analyzed by electrophoresis and Western blotting using anti-recombinant *L. laeta* PLD1 serum. Phospholipase D enzymatic activity was assessed, and the hemolytic and cytotoxic effects were evaluated and compared with those of the *L. laeta* venom. The *S. thomisoides* venom was able to hydrolyze sphingomyelin as well as induce complement-dependent hemolysis and the loss of viability of skin fibroblasts with a dermonecrotic effect of the venom in rabbits. The venom of *S. thomisoides* showed intraspecific variations, with a similar protein pattern as that of *L. laeta* venom at 32–35 kDa, recognized by serum anti-LlPLD1. In this context, we can conclude that the venom of *Sicarius thomisoides* is similar to *Loxosceles laeta* in many aspects, and the dermonecrotic toxin present in their venom could cause severe harm to humans; thus, precautions are necessary to avoid exposure to their bite.

## 1. Introduction

The spider family called *Sicariidae* includes the genera *Loxosceles*, *Sicarius,* and, recently, *Hexophthalma* [1]. All the spider species in this family are considered potentially harmful to humans [2]. However, while the *Loxosceles* spiders are thoroughly documented as a cause of envenomation in humans, called Loxoscelism [3,4], little information is available about the toxic potential of *Sicarius* spiders.

The distinctive morphology of *Loxosceles* includes a brown-colored body with a dark, violin-shaped mark on the cephalothorax. It also has three pairs of eyes arranged in a U-shaped pattern, with sexual dimorphism; females have larger abdomens, and males have palps with a specialized structure called the spermophore [3]. Envenomation by *Loxosceles* is characterized by local lesions, with erythema, edema, inflammation, and dermonecrosis as well as systemic reactions such as hemolysis, renal failure, and hematologic alterations that include thrombocytopenia, disseminated intravascular coagulation, and hemolytic anemia [4,5,6].

The genus *Sicarius* [7] is larger and more aggressive than *Loxosceles*. These spiders inhabit desert and semidesert climates and are known as six-eyed sand spiders regarding their habit of covering up and burying themselves with sand [8,9]. Several species of *Sicarius* have been reported in Chile. Among them, the species *Sicarius thomisoides* (Walckenaer, 1847) [7] has a wide distribution, from the desert biomes of the Atacama Desert to central zones [8]. These spiders have nocturnal habits, so they shelter under large and warm rocks during the day [9]. This species is larger than others of the same genus in Chile, measuring between 12 and 20 mm in body length (not including the legs). Currently, it is the only known species that can bite and attack vertebrates [10].

*Sicarius* and *Loxosceles* share the presence of phospholipase D (formerly sphingomyelinase D) in their venoms, in contrast to other Haplogyne spiders [11,12]. The phospholipase D (PLD) family of toxins is the most studied and well-known component of *Loxosceles* venom, and both native and recombinant PLDs alone can reproduce all the clinical symptoms of Loxoscelism [13]. Although the presence and activity of PLDs have been demonstrated in some species of *Sicarius* from Africa (now considered as *Hexophthalma* genus), less has been studied about the toxic potential in South American *Sicarius* venom to cause a pathology resembling that caused by *Loxosceles* venom. Only one previous study on the Brazilian spider *Sicarius ornatus* demonstrated the active presence of sphingomyelinase D, as well as hemolytic and cytotoxic activities of the venom [14]. Here, we characterized the venom of the Chilean spider *Sicarius thomisoides*, demonstrating the presence of active phospholipase D, by comparing the hemolytic and cytotoxic activities with those of *L. laeta* and reporting the venom capacity to produce a dermonecrotic lesion.

## 2. Results

### 2.1. Description of the Captured S. thomisoides and Determination of the Venom Protein Concentration

**Figure 1:**
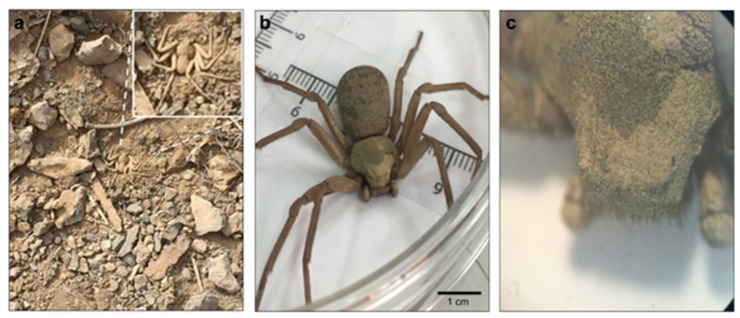
*Sicarius thomisoides* spider. (**a**) *S. thomisoides* in its natural habitat in La Chimba National Park, Antofagasta, Chile. (**b**) Female *S. thomisoides*. (**c**) Magnification (2×) of *S. thomisoides* cephalothorax. Scale bar: 1 cm.

*Sicarius thomisoides* spiders were captured in semidesert areas, where they live under rocks (Figure 1). Sixteen *S. thomisoides* specimens were captured, three of which were identified as females, while no males were captured. The rest of the thirteen specimens captured were identified in the nymph stage (immature or juveniles). *S. thomisoides* spiders are morphologically characterized by body pigmentation between bone-white to a light brown or a type of “earthy brown” and the presence of mace-shaped body macrosetes with combs to which soil particles can adhere, covering the dorsal surface of their carapace, opistosome, and legs. Their body size ranged from 15 to 50 mm long, including the legs [7]. Thus, all the captured *S. thomisoides* showed the characteristic light brown pigmentation including the soil particles adhered on their body (Figure 1). Because spiders of the *Sicarius* genus belong to the *Sicariidae* family, where the *Loxosceles* genus is also included [1], we first compared the body length and weight of the captured *S. thomisoides* spiders with the *L. laeta*. The captured females of *S. thomisoides* were three times larger than those of *L. laeta*. In *S. thomisoides* females, the body length ranged from 2 to 3 cm up to 5 to 8 cm, including their legs, and they weighed between 0.5 and 1.0 g. However, *L. laeta* females showed a size ranging from 1 to 1.5 cm in body length up to 4–5 cm in body length, including the legs, and they weighed between 0.03 and 0.4 g (Table S1). Additionally, length and weight differences were observed between nymphs of *S. thomisoides*; thus, they were classified as small nymphs or early nymphs (first to sixth nymphal stage; size= 0.5–0.7 cm in body length, and 3 to 3.5 cm in body length including the legs; the weight ranged between 0.06 and 0.19 g) and larger nymphs or those in the later nymphal stage (sixth to ninth nymphal stage; size= 1.5–2 cm in body length and 3.5–4 cm in body length including the legs; the weight ranged between 0.2 and 0.4 g). Additionally, the larger nymph stage specimens of *S. thomisoides* showed a size similar to that of the *L. laeta* adults (Table S1).

**Figure 2:**
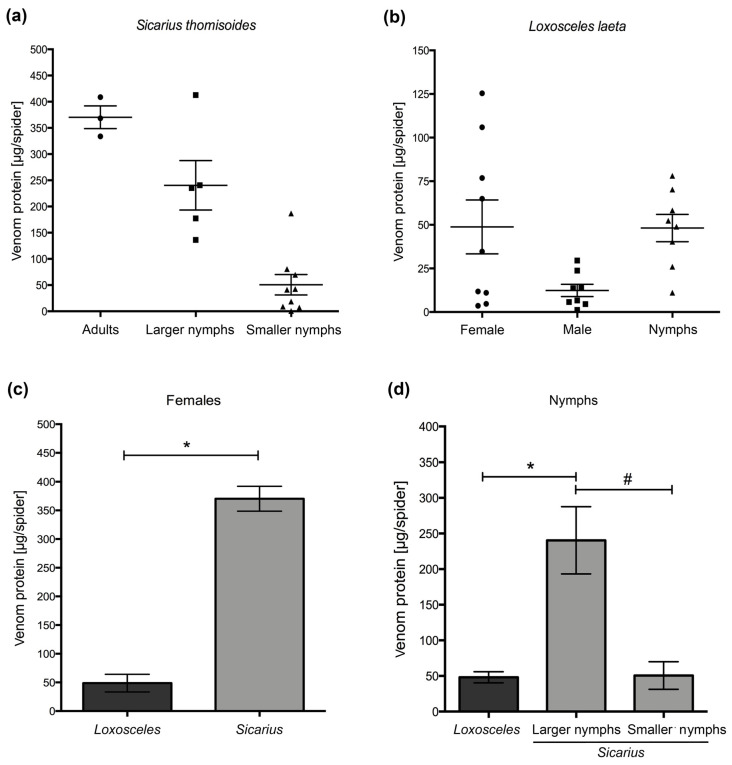
*S. thomisoides* venom protein quantitation. (**a**) Venom protein (μg/spider) distribution of *S. thomisoides* adults (*n* = 3), larger nymphs (*n* = 5), and smaller nymphs (*n* = 9). (**b**) Venom protein (μg/spider) distribution of *Loxosceles laeta* in adults: female (*n* = 9) and male (*n* = 8) and nymphs (*n* = 8). (**c**) Comparison of the venom protein (μg/spider) between *S. thomisoides* and *L. laeta* female specimens. (**d**) Comparison of the venom protein (μg/spider) between *S. thomisoides* and *L. laeta* nymph specimens. The results were expressed as the means ± SD. (*) Statistical significance (*p* < 0.05) between venom protein means of females from *S. thomisoides* and *L. laeta*. (#) Statistical significance (*p* < 0.05) between the venom protein means of *L. laeta* nymphs and *S. thomisoides* small nymphs.

The amount of spider venom (µg/spider) for the different specimens of *S. thomisoides* increased according to the size, weight, and development stage (from nymphs to adults) (Figure 2a), with a mean value ± standard deviation (SD) of 370.3 ± 37.49 µg/spider and a median of 368.3 µg/spider for females, a mean value ± SD of 240.4 ± 105.5 µg/spider and median of 235.4 µg/spider for large nymphs, and mean value ± SD of 50.66 ± 58.23 µg/spider and median of 41.18 µg/spider for small nymphs. However, the venom protein concentrations (µg/spider) of *L. laeta* adult specimens (female and male) and nymphs were similar (Figure 2b). The mean and median values were as follows: 48.8 ± 46.3 µg/spider and 34.7 µg/spider, respectively, for females; 12.4 ± 9.9 µg/spider and 10.1 µg/spider, respectively, for males; 48.2 ± 22.1 µg/spider and 50.7 µg/spider, respectively, for nymphs. Additionally, the protein yield in the venom of *S. thomisoides* females was 7.7 times higher than that in female *L. laeta* venom (Figure 2c). Additionally, the venom protein yield of *S. thomisoides* large nymphs was 5 times higher than that of *L. laeta* females (Figure 2d). However, the venom protein yield of *S. thomisoides* small nymphs showed no differences with those of the venom proteins of *L. laeta* females.

### 2.2. Electrophoretic Characterization of Venom from the S. thomisoides Spider and Evaluation of Phospholipase D activity

**Figure 3:**
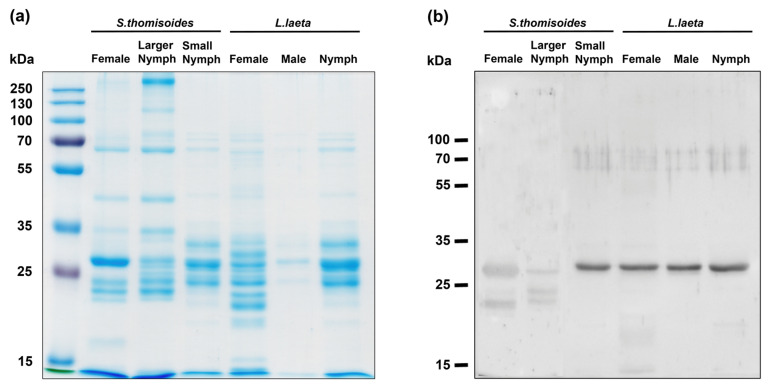
Electrophoretic pattern of *S. thomisoides* venom and phospholipase D detection by Western blotting. (**a**) Venoms (10 µg) in the females and nymphs of *S. thomisoides* were subjected to 12% SDS-PAGE and compared with 10 µg of venom from females, males, and nymphs of *L. laeta*. (**b**) The venoms were detected by Western blotting using mouse polyclonal serum against rLlPLD1 from *L. laeta* diluted 1:1000, followed by anti-mouse IgG (H + L)-HRP conjugate diluted 1:40,000 and developed using electrochemiluminescence (ECL).

**Figure 4:**
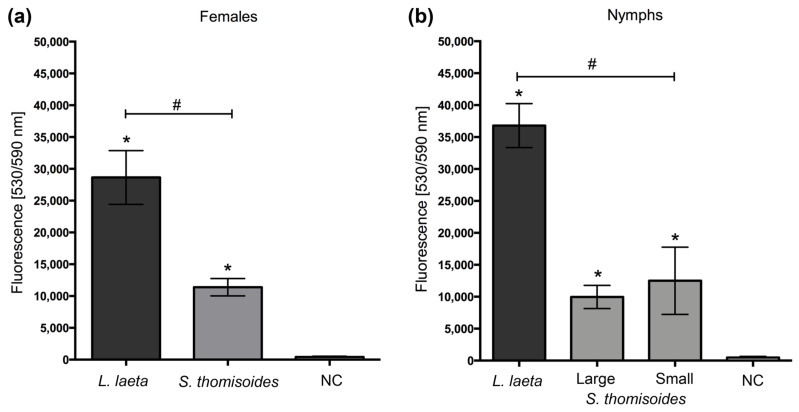
Comparison of phospholipase D activity between the *S. thomisoides* and *L. laeta* venoms. (**a**) Female venoms. (**b**) Nymphs venoms. The results were expressed as the means ± SEM. (*) Statistical significance (*p* < 0.05) between the means of *L. laeta* or *S. thomisoides* venoms compared with the negative control (NC). (#) Statistical significance (*p* < 0.05) between the means of *L. laeta* and *S. thomisoides* venoms.

The spider venom was analyzed by SDS-PAGE, showing differences in the number of protein bands of venoms from females and nymphs of *S. thomisoides* and in comparison with females, males, and nymphs from *L. laeta* (Figure 3a). However, a similar protein band pattern was detected between 32 and 35 kDa. To assess whether the venom from the *S. thomisoides* spider has phospholipase D, as well as the one found in the band range of 32–35 kDa in *L. laeta*, the venoms were analyzed by Western blotting using polyclonal serum raised against a recombinant isoform phospholipase D1 from *L. laeta* (rLlPLD1). As shown in Figure 3b, the polyclonal antibodies recognized a single band of the venom from *L. laeta* and *S. thomisoides*. However, the detection of *S. thomisoides* PLDs was lower in larger nymphs or females than in the small nymphs. To corroborate whether the bands recognized by pAb-anti-rLlPLD1 in the venom from *S. thomisoides* were phospholipase D, we measured the ability of phospholipase D in the venom of females and nymphs of *S. thomisoides* and *L. laeta* to hydrolyze sphingomyelin. As shown in Figure 4, the venom from females and nymphs of *S. thomisoides* showed significant sphingomyelinase D activity compared with the negative control (assay buffer). However, these activities were significantly lower than sphingomyelinase D activity in females and nymphs of *L. laeta*. Thus, the presence and activity of phospholipase D in the venom of *S. thomisoides* spiders were demonstrated.

### 2.3. The Venom of S. thomisoides Spiders Induces Hemolysis, Cytotoxicity, and Dermonecrosis

**Figure 5:**
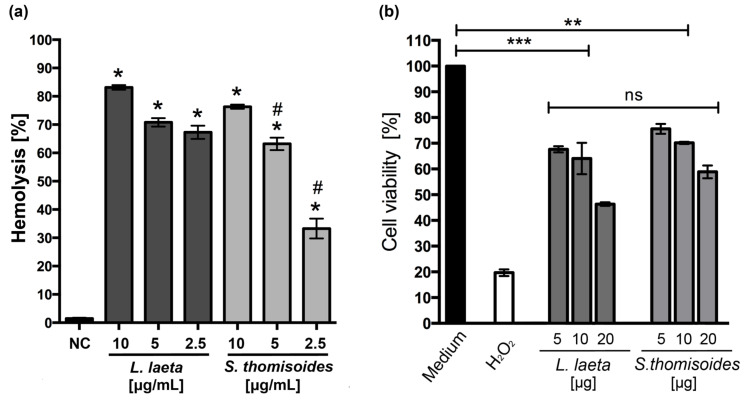
Comparison of complement-dependent hemolytic and cytotoxic activities between the venoms of *S. thomisoides* and *L. laeta*. (**a**) Complement-dependent hemolysis of human erythrocytes treated with 10, 5, and 2.5 µg/mL of pooled venoms of females from *S. thomisoides* and *L. laeta*. The hemolysis absorbance was read at 414 nm and expressed as the hemolysis percentage of three different experiments in triplicate (mean ± SEM). NC: negative control. (*) Statistical significance (*p* < 0.05) between the means of *S. thomisoides* or *L. laeta* venoms from the negative control. (#) Statistical significance (*p* < 0.05) between the means of *S. thomisoides* and *L. laeta* venoms. (**b**) Viability assay of HFF-1 skin fibroblasts to test the cytotoxic activity of pooled venom of females from *S. thomisoides* and *L. laeta* venoms. The cells were treated for 24 h at 37 °C with 5, 10, and 20 µg of venoms from *S. thomisoides* and *L. laeta*. The control of 100% viability was treated only with the medium. The results were expressed as the viability percentage against the control of 100% (medium) in two experiments in triplicate (mean ± SEM). ns: no statistical significance. (*) Statistical significance (*p* < 0.05) between the means of *S. thomisoides* or *L. laeta* venoms compared with the control (100% viability). **, *p* < 0.01, ***, *p* < 0.001.

**Figure 6:**
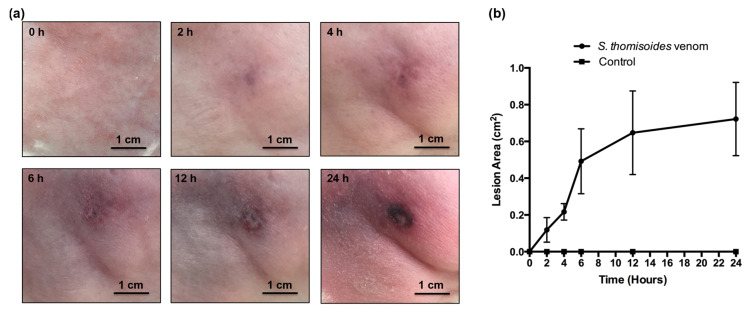
Dermonecrosis assay of *S. thomisoides* venom. (**a**) The pooled venom of females of *S. thomisoides* (50 µg) was inoculated into the rabbit skin, and a lesion was recorded between 2 and 24 h post inoculation. Scale bar = 1 cm. (**b**) The lesion areas (cm^2^) from three treated rabbits were measured and graphed. Control: PBS-treated rabbits.

To assess whether the venom of *S. thomisoides* has toxic activity similar to that of the *L. laeta* spider, we compared the hemolytic and cytotoxic activities of both venoms. As shown in Figure 5a, the venom of *S. thomisoides* produced complement-dependent hemolysis in a concentration-dependent way, similar to *L. laeta* venom at 10 µg/mL. Additionally, the venom from *S. thomisoides* showed significant cytotoxic activity against HFF-1 human skin fibroblasts after 24 h of treatment with 5, 10, and 20 µg of venom. No significant differences were found with the cytotoxic activity of the venom from *L. laeta* (Figure 5b). Finally, the ability of the *S. thomisoides* venom to produce dermonecrosis in the skin of rabbits was assessed. As shown in Figure 6, the venom of *S. thomisoides* (50 µg) produced dermonecrosis progressively over time (2 to 24 h), forming a necrotic lesion surrounded by erythema and marked edema. However, when 10 µg of StV was used, only erythema and edema were observed at 24 h (Figure S1). Therefore, the venom of the *S. thomisoides* spider fulfills the toxic parameters to cause harm to humans.

## 3. Discussion

A spider is considered potentially dangerous for humans when the spider has venom with documented pathogenic activity for humans. Additionally, it must live where humans live or carry out their activities (synanthropism), as well as possess chelicerae capable of perforating the skin and injecting its venom [15]. In Chile, synanthropic spiders that are considered as dangerous include those from the *Sicariidae* and *Theridiidae* families, which are represented by the *Loxosceles laeta*, *Latrodectus,* and *Steatoda* species [16]. However, little attention has been given to nonsynanthropic spiders, such as those of the *Sicarius* species. Therefore, we studied the venom toxicity of *S. thomisoides*, which has been documented as one of the largest and most aggressive spiders in the genus *Sicarius* in Chile [8,9]. These spiders are poisonous and prey on insects and occasionally vertebrates [10]. This background makes them an ideal model to study the potential toxicity of this spider’s venom. Thus, we were able to capture some individuals of the Chilean spider *S. thomisoides* from desert and coastal locations of the Atacama Desert, in the north of Chile, which were quite nearby to human urban settlements. Unfortunately, only few adults were captured, and all of them were females (*n* = 3). In this regard, the presence of adults in the populations of this species is quite rare, since they seem to be long-lived spiders that take a long time to reach adulthood [8], as was shown by the number of larger nymphs (sixth to ninth nymph stages) captured, which could easily be confounded with adults if properly sexual characterization is not performed. In addition, due to the time of year when spiders were captured (late summer to late fall), we were unable to find adult males since, as ectothermic organisms, the microhabitat selection is limited by the thermal conditions of their environment [9]. Furthermore, the frequency of males from *S. thomisoides* spiders has been reported to be less than 3% of the spider population in different coastal and inland locations in the north of Chile [9]. However, a comparative sex-linked study with more adult specimens seems to be necessary to determine the toxic potential of venoms from males and females of *S. thomisoides* spiders, based on the results shown here, and also to accurately correlate the morphology and size differences with the venom protein yield.

Thus, our data demonstrated that *S. thomisoides* spiders possess a higher venom protein yield (µg/spider) and venom volume than *L. laeta*. These features are particularly interesting because *L. laeta* have been considered as the one of the *Loxosceles* species, with the highest protein concentration and most dangerous venom [17,18]. Thus, we focused on comparing the biochemical and biological properties of venoms between *S. thomisoides* and *L. laeta*. The differences in the venom volume and concentration between the spiders could be explained by size differences between the spiders and those of their venom glands (since *S. thomisoides* is bigger than *L. laeta*) [10]. Thereby, we found that *S. thomisoides* adults were three times larger in size than *L. laeta* adults. Similar differences in the venom concentration between *Sicarius* and *Loxosceles* were also reported for females and males in the *Sicarius ornatus* species compared with the females of *L. laeta*. However, no comparison was performed between the nymph stage of both spider genera [14]. Here, we showed marked differences in the venom concentration for the later stages of development of *S. thomisoides* compared with any nymph stage of *L. laeta*. The cause may be related to the spider’s body size because small individuals of *S. thomisoides* with a similar size to that of any nymph stage of *L. laeta* had the same concentration of venom as that of *L. laeta*. Moreover, prey selection could also influence the differences in venom volume. Regarding this, spiders with a generalist prey diet have been reported to have large-sized venom glands, a large venom volume, and more complex venom than spiders with specialized prey selection that have smaller glands and less complex venom [19]. Thus, both genera, *Sicarius* and *Loxosceles*, which belong to the *Sicariidae* family, can be considered spiders with a generalized prey selection [19]. Therefore, in addition to the spider size differences, the significant contrast in the venom volume and protein concentration observed could be related to the adaptation of prey present in their habitat. *Loxosceles* inhabits intradomiciliary environments, whereas *Sicarius* inhabits extradomiciliary environments where prey diversity is higher; thus, *Sicarius* can prey on many epigeal insects [8] as well on smaller vertebrates such as the gecko *Phyllodactylus gerrhopygus* [10].

The variations in the electrophoretic pattern of the venom from *S. thomisoides* shown between adult and nymph venom were consistent with intraspecific variations. These intraspecific variations were also reported for the venoms of female and male *S. ornatus* [14], as well as for the venoms of *Loxosceles* species, including *L. laeta* [17,18]. Additionally, regardless of the similarities between the protein profiles of venom from *S. thomisoides* and *L. laeta*, we observed differences between the electrophoretic pattern of venoms, indicating interspecific variations. However, both spider venoms showed similar protein bands in the range of 25 to 35 kDa. In *Loxosceles* venom, the protein components between 25 and 35 kDa have been characterized as members of the phospholipase D family, and they are present in different *Loxosceles* species [11]. Thus, the protein bands of *S. thomisoides* venom in this range could be considered as paralog PLD enzymes of those present in *Loxosceles* venom [2,20]. Therefore, we used polyclonal anti-recombinant PLD from *L. laeta* serum to detect potential PLDs in *S. thomisoides* venom; a single band was recognized, confirming the presence of PLDs in the venom. This detection was higher in the nymph venom than in the adult venom, likely because of the monospecific characteristics of the serum we used and the differences between the development stages of the spider, where nymphs could be considered more active than adults, consequently expressing more PLD. Additionally, we cannot rule out that Western blotting differences were the consequence of antigenic differences between PLDs from *Sicarius* and *Loxosceles*. However, recently, we reported that natural-produced and specific-produced antibodies against *L. laeta* venom could cross-detect the venom from *Sicarius* spiders [21], corroborating the similarities between the protein components of venoms from *S. thomisoides* and *L. laeta*. Additionally, polyclonal serum against *L. intermedia* SMase D has been reported to detect the *S. ornatus* protein band at 32–35 kDa, confirming that venom proteins in this protein range were PLDs [14].

The presence of PLD activity in the venom of *S. thomisoides* is critical to consider this venom toxic to humans, as all recombinant PLDs from *Loxosceles* that possess *in vitro* sphingomyelinase activity can trigger the local and systemic effects observed for the venom [13]. *Loxosceles* PLD activity has been reported to be associated with the hydrolysis of sphingomyelin to form ceramide 1-phosphate (C1P) and choline, as well as the hydrolysis of lysophospholipids such as lysophosphatidylcholine to form lysophosphatidic acid (LPA) and choline [22,23,24,25]. This sphingomyelinase D activity has been demonstrated in different African and American *Sicarius* species, with higher activity in African than in American species [2]. Due to the above findings, we evaluated the phospholipase D activity in *S. thomisoides* venom against the sphingomyelin substrate, observing that the venom possesses phospholipase D activity, but with differences in the activity at the intraspecies level (at the stages of development, from nymphs to adults). This finding was also confirmed by Western blot analysis. However, the phospholipase D activity of *S. thomisoides* venom was significantly lower than that of *L. laeta* venom. These differences were also shown in the adults of *S. ornatus* compared with the activity in the venoms of *L. laeta* and *L. gaucho* [14]. The cause may be the differences in the number of isoforms present in the venom of *Sicarius* compared with that in the venom of *Loxosceles*. Indeed, the differences in the number of isoforms between the species of *Loxosceles* spiders have been documented [13,26,27].

Furthermore, differences in the tertiary structure of *Sicarius* PLD compared with that of PLDs of *Loxosceles* could affect the hydrolysis capacity of the enzyme. In this regard, two classes of phospholipase D (class I and class II) have been documented in the venom of *Loxosceles*, with differences in their tertiary structure and the presence of a single disulfide bridge and extended hydrophobic loop (class I), or an additional intrachain disulfide bridge linked to a flexible loop and catalytic loop (class II), which are further subdivided into more active isoforms (classes IIa) and less active or inactive isoforms (classes IIb) [28,29]. Subsequent studies at the proteomic level are required to respond to the above, either through purification of the enzyme, followed by three-dimensional structure identification by crystallography, or by the cloning and expression of recombinant isoforms of PLDs from *Sicarius*, followed by three-dimensional structure prediction using 3D modeling.

The pathophysiological effects of *Loxosceles* venom associated with the activity of PLDs include complement-dependent hemolysis, cytotoxicity, and dermonecrosis [23,30]. To assess whether the *S. thomisoides* venom could produce the three key aspects shown for *Loxosceles* venom, human erythrocytes were incubated with *S. thomisoides* or *L. laeta* venom and compared. The hemolytic effect of *S. thomisoides* venom was similar to that of *L. laeta* venom, and only less hemolysis was shown when a lower concentration was assayed. The venom of the *S. ornatus* species also showed complement-dependent hemolytic activity, which was induced by glycophorin C cleavage after the binding of SMase-D to the erythrocyte membrane [14]. Whether *S. thomisoides* complement-dependent hemolytic activity was a consequence of the same mechanism as that for the venom of *S. ornatus* remains to be elucidated. Nevertheless, it is highly possible that both species of South American *Sicarius—S. ornatus* and the *S. thomisoides*—share the same hemolytic mechanism due to the results shown here and shown for *Loxosceles* species [31]. Moreover, *S. thomisoides* venom showed cytotoxicity to human skin HFF-1 fibroblasts, without significant differences compared with *L. laeta* venom. However, the effect of *S. thomisoides* venom on the viability of dermal human skin fibroblasts was early (24 h) and higher than that shown by *S. ornatus* venom on HaCaT human epidermal keratinocytes, which showed reduced viability only after 48 to 72 h of treatment [14]. Although the differences could be explained by the interspecific variations of the venoms, we cannot rule out that the *Sicarius* venom has a different effect depending on the skin cell type (dermal over epidermal) and availability of sphingomyelin in cell membranes because of the differences in the regulation of lipids, as was shown for cholesterol synthesis between human skin fibroblasts and keratinocytes [32]. Finally, we verified that one of the largest species of *Sicarius* present in Chile was able to produce dermonecrosis on rabbit skin. Few reports have shown dermonecrotic lesions caused by *Sicarius* spiders. Most have been associated with the African species *Sicarius testaceus* [33]; however, this specie is now considered as member of the *Hexophthalma* genus, as well as others African *Sicarius* [34]. Thus, comparative dermonecrosis studies between genera are required to determine possible differences at the genera level. However, the ability of South American *Sicarius* to produce dermonecrosis has been demonstrated in a single report documenting a human bitten by the South American *Sicarius tropicus* who developed a necrotic lesion [35]. In our dermonecrosis assay we demonstrated that 50 µg of *S. thomisoides* venom was able to produce dermonecrosis in rabbits. However, a lower dose (10 µg) failed to produce dermonecrosis. The venom concentration of 50 µg of *S. thomisoides* venom seems to be enough to produce dermonecrosis. Thus, higher doses of venom inoculated by adults of *S. thomisoides* could cause more serious lesions, since adults posses up to eight times the concentration assayed. These concentrations correlate with the range of venom able to produce dermonecrosis in rabbits by *L. intermedia* venom, where 20 µg of venom was demonstrated as the lower dose able to produce dermonecrosis, with an average of 40 µg of venom [36,37].

In conclusion, because the venom of *S. thomisoides* has phospholipase D activity and can cause hemolysis, cell death of skin fibroblasts, and dermonecrosis, it fulfills the requirements to be considered a spider able to produce harmful effects to humans. Therefore, *S. thomisoides* must be included and considered a dangerous spider in Chile, and precautions must be taken to avoid human exposure to this spider.

## 4. Materials and Methods

### 4.1. Spiders and Venoms

Sixteen *Sicarius thomisoides* spiders were captured in desert and semidesert areas such as La Chimba National Park and Juan Lopez Bay, in the Antofagasta Region, as well as in La Huayca, in the Tarapacá Region; both regions are located in northern Chile and close to urban settlements where humans live. Additionally, twenty-five *Loxosceles laeta* spiders were captured from domiciliary habitats in the city of Antofagasta. Among them, nine specimens were classified as females, eight as males, and eight as nymphs or immatures. All the spiders were maintained at the Molecular Parasitology Research Laboratory at the University of Antofagasta, Chile. However, the spider identification was performed at the Environmental Research Center (CENIMA) at the Arturo Prat University in Iquique based on the morphological characteristics of the spiders. The *L. laeta* spider was identified using the morphological characteristics reported by Gertsch (1967) [38]; the specimens were then classified according to stages in adults (males and females) and nymphs. *S. thomisoides* spiders were identified using morphological characteristics reported by Magalhaes et al. (2017) [8]; the specimens were classified according to stages in adults or nymphs. Nymphs were subclassified as small nymphs (from first to sixth nymph stages) and large nymphs (from sixth to ninth nymph stages) based on their body size and weight differences.

*S. thomisoides* (Stv) and *L. laeta* venom (Llv) were extracted by electrostimulation from the spiders after a week of captivity and with no previous feeding. Then, the venom droplets were collected with a micropipette in 30 μL of PBS and stored at −80 °C until use, as previously reported [39]. The protein concentration of venom samples was evaluated by the Bradford dye-binding method [40] using the Bio-Rad Protein Assay kit (Bio-Rad Laboratories, Inc., Hercules, CA, USA), and the amount of protein in μg by spider was calculated based on the volume of venom collected.

All the protocols for the biological research in invertebrate and/or biotechnological species, including the procedures for spider capture, spider maintenance, and venom extraction, were approved by the Ethics Committee in Scientific Research of the University of Antofagasta (CEIC-UA) (CEIC-REV No. 06/2019).

### 4.2. Electrophoresis and Immunoblotting

Ten micrograms of venom samples from females, large and small nymphs of *S. thomisoides,* and both adults (male and female) and nymphs of *L. laeta* spiders was electrophoretically separated on 12% SDS-PAGE gels under nonreducing conditions and stained with Coomassie blue G-250. Next, the gels were transferred to nitrocellulose membranes. After transfer, the membranes were blocked for 2 h with 5% nonfat milk in TBS/0.1% Tween20 (TBS-T) and incubated for 1 h at room temperature with polyclonal mouse anti-rLlPLD1 serum (1:1000 dilution) produced previously [41]. The membranes were washed six times for 10 min each with TBS-T and incubated with anti-mouse IgG (H + L)-HRP conjugate (1:40,000 dilution) in TBS-T for 1 h at room temperature. After another six washes with TBS-T, the membranes were developed using the ECL^TM^ Western blotting detection reagent kit (GE Healthcare, Piscataway, NJ, USA). Mouse polyclonal anti-*L. laeta* recombinant phospholipase D1 (rLlPLD1) was prepared as previously documented [41].

### 4.3. Phospholipase D Activity

The phospholipase D activities of *S. thomisoides* or *L. laeta* venom toward the sphingomyelin substrate were measured using the Amplex Red Sphingomyelinase D Assay kit (Molecular Probes, Eugene, OR) following the manufacturer’s instructions [41]. In summary, 2.5 μg/mL of venom from the *S. thomisoides* and *L. laeta* females and nymphs was incubated with sphingomyelin in Amplex Red reaction buffer at 37 °C for 1 h, and the fluorescence was measured using an Infinite M200 PRO microplate reader (Tecan) spectrofluorometer and excitation/emission filters of 530 and 590 nm, respectively. The assays were performed in duplicate for three separate experiments. The fluorescence was compared against the negative control (NC) using only the Amplex Red reaction buffer.

### 4.4. Complement-Dependent Hemolysis Assay

The human erythrocyte complement-dependent hemolysis assay was performed as previously described [41]. Briefly, human erythrocytes were washed three times with Veronal Buffered Saline (VBS; pH 7.4, 10 mM sodium barbitone, 0.15 mM CaCl_2_, 0.5 mM MgCl_2_, and 145 mM NaCl) and resuspended at 2% in VBS. The cells were sensitized for 30 min at 37 °C with 10, 5, or 2.5 μg/mL of pooled venom from the females of *S. thomisoides* or *L. laeta* in 100 μL of VBS. After incubation, the sensitized erythrocytes were washed three times with VBS and analyzed by the complement-dependent hemolysis assay. Next, 100 μL of sensitized erythrocytes was mixed with 100 μL of normal human serum (NHS; 1:2 in VBS). The negative control was evaluated by incubating the erythrocytes with VBS, while the total hemolysis control was incubated with H_2_O. After incubation for 1 h at 37 °C, the nonlysed cells were centrifuged at 440× *g* for 5 min, the supernatant was collected, and absorbance was measured at 414 nm. The results were expressed as a percentage of hemolysis and calculated based on the absorbance of the 100% hemolysis control. The assays were performed in duplicate for two independent experiments. The erythrocytes and normal serum were obtained from the same donor.

### 4.5. Viability Assay

The viability assay was performed as previously described [42]. The cultures of HFF-1 human skin fibroblasts with more than 95% viability were used for the viability assay. Briefly, a cell suspension of 2 × 10^4^ cells/wells in DMEM serum-free medium was placed in each well of a 96-well culture plate. Subsequently, the cells were incubated for 4 h at 37 °C in a humidified atmosphere containing 5% CO_2_, allowing cell adherence. Next, 40 µg of pooled venom from the adults of *S. thomisoides* or *L. laeta* was two-fold diluted in serum-free DMEM medium and was added to the cells, followed by incubation at 37 °C for 24 h under an atmosphere containing 5% CO_2_. Untreated cells were used as a 100% viability control (negative control), while cells treated with 0.3% hydrogen peroxide (H_2_O_2_) were used as a positive mortality control. Next, the cell viability for each treatment was determined using Cell Titer 96 Aqueous One Solution (Promega) according to the manufacturer’s instructions, in which 20 µL of reagent was dispensed to each well and incubated at 37 °C for 1 h. Subsequently, viable cells were measured at 490 nm in an Infinite M200 PRO microplate reader (Tecan Group Ltd., Männedorf, Switzerland). The percentage of viability was calculated as follows: % viability = (Abs490 nm sample—Abs490 nm blank control)/(Abs490 nm control 100% viability—Abs490 nm blank control) × 100. Additionally, the inhibitory concentration 50 (IC50) for each treatment was calculated using nonlinear regression on a sigmoidal curve. Assays were performed in triplicate for two independent experiments.

### 4.6. Dermonecrosis Assay

Groups of three New Zealand male rabbits were inoculated intradermally, into a previously shaved right leg, with 10 µg or 50 µg of pooled venom from the females of *S. thomisoides*. Next, the presence of erythema, edema, or necrosis was documented by photography of the inoculated area at 2, 4, 6, 12, and 24 h after inoculation. Sterile PBS inoculated into the left leg of rabbits was used as negative control. The lesion area (cm^2^) of the region of interest (ROI) for each dermonecrotic lesion was analyzed using ImageJ software (National Institutes of Health, Bethesda, MD, USA) and graphed [43].

### 4.7. Statistical Analysis

One-way ANOVA followed by Tukey’s multiple comparisons test was performed using GraphPad Prism software v.6.0. A significant criterion of *p*-value < 0.05 was used.

## Supplementary Materials

The following are available online at https://www.mdpi.com/2072-6651/12/11/702/s1: Table S1: Characteristic of captured *Sicarius thomisoides* specimens (development stage, sex, weight, venom volume extracted, and venom protein yield by spider); Figure S1: Dermonecrosis assay of *S. thomisoides* venom using 10 µg of venom inoculated into rabbit skin.
