# Supplementary material for: Toxicological Characterization and Phospholipase D Activity of the Venom of the Spider Sicarius thomisoides
Source: Toxins (Basel). 2020 Nov 6;12(11):702. doi: 10.3390/toxins12110702 (PMC7694614; doi:10.3390/toxins12110702)
Supplement: Supplementary file 1 [file toxins-12-00702-s001.pdf]

Supplementary Materials: Toxicological characterization and Phospholipase-D activity of venom from the spider *Sicarius thomisoides*

Tomás Arán-Sekul, Ivanka Perčić-Sarmiento, Verónica Valencia, Nelly Olivero, José M. Rojas, Jorge E. Araya, Andrés Taucare-Ríos and Alejandro Catalán

Table S1. Characteristics of captured and studied spiders (development stage, sex, weight, size, venom volume extracted, and venom protein yield by spider).

Stage	Sex	Spider ID	Weight (g)	Size (cm) Body Length; Body Length Including Legs	Extracted Venom Volume (µL)	Extracted Venom Protein Concentration (µg/µL)	Venom Protein Yield by Spider (µg/Spider)
Adults	Females	StF1	1	3; 8	4.5	10.68	368.34
		StF2	0.55	2; 5	3	10.12	333.89
		StF3	0.73	2.5; 6.8	8	10.76	408.79
	Males	NC	ND	ND	ND	ND	ND
Nymphs	Large	StLN1	0.37	2; 4	6.5	4.85	177.19
		StLN2	0.29	1.5; 3	3	12.5	412.58
		StLN3	0.29	1.5; 3.5	5.5	3.84	136.32
		StLN4	0.31	2; 3	5.5	6.63	235.37
		StLN5	0.23	1.5; 3.2	2	7.52	240.64
	Small	StSN1	0.12	0.7; 3	1.5	5.93	186.8
		StSN2	0.1	0.5; 3.2	1.5	2.56	80.64
		StSN3	0.11	0.6; 3	0.9	0.29	8.96
		StSN4	0.14	0.7; 3.5	0.3	0.23	6.97
		StSN5	0.09	0.5; 3	1.2	1.32	41.18
		StSN6	0.09	0.65; 3	0.7	2.27	69.69
		StSN7	0.06	0.5; 2.9	1	1.37	42.47
		StSN8	0.07	0.6; 3	0.1	0.62	18.66
*L. laeta**							
Adults	Females (*n*=9)		0.15	1-2 ; 4-5	0.82	1.56	53.49
	Males (*n*=8)		0.15	1-1.3; 4-6.5	0.16	0.41	12.36
Nymph	(*n*=8)		0.09	0.5-1; 2.5-3	0.49	1.58	48.17

* *L. laeta* values correspond to average of specimens of each stage. StF: *S. thomisoides* female. StLN: *S. thomisoides* large nymph. StSN: *S. thomisoides* small nymph. NC: Not Captured. ND: No data.

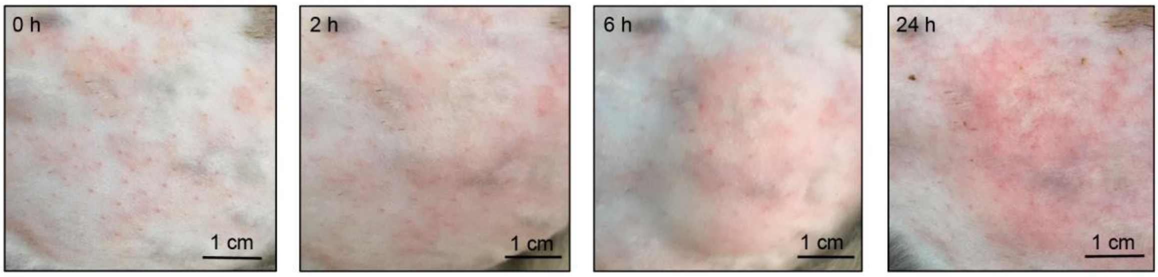

Figure S1. Dermonecrosis assay of *S. thomisoides* venom using 10 µg of venom inoculated into rabbit skin. Lesions were recorded between 2 at 24 h post inoculation. Scale bar = 1 cm. The images show apparitions of edema and erythema only, and no dermonecrosis was observed.
